# Identifying the translational complexity of magnetic resonance spectroscopy in neonates and infants

**DOI:** 10.1002/nbm.4089

**Published:** 2019-03-29

**Authors:** Hunter G. Moss, Dorothea D. Jenkins, Milad Yazdani, Truman R. Brown

**Affiliations:** ^1^ Department of Radiology Medical University of South Carolina Charleston South Carolina; ^2^ Department of Pediatrics Medical University of South Carolina Charleston South Carolina

**Keywords:** hypoxic–ischemic encephalopathy, magnetic resonance spectroscopy, neonates, premature infants

## Abstract

Little attention has been paid to relating MRS outputs of vendor‐supplied platforms to those from research software. This comparison is crucial to advance MRS as a clinical prognostic tool for disease or injury, recovery, and outcome.

The work presented here investigates the agreement between metabolic ratios reported from vendor‐provided and LCModel fitting algorithms using MRS data obtained on Siemens 3 T TIM Trio and 3 T Skyra MRI scanners in a total of 55 premature infants and term neonates with hypoxic ischemic encephalopathy (HIE). We compared peak area ratios in single voxels placed in basal ganglia (BG) and frontal white matter (WM) using standard PRESS (TE = 30 ms and 270 ms) and STEAM (TE = 20 ms) MRS sequences at multiple times after birth from 5 to 60 days.

A total of 74 scans met quality standards for inclusion, reflecting a spectrum of neonatal disease and several months of early infant development. For the long TE PRESS sequence, N‐acetylaspartate (NAA) and Choline (Cho) ratios to Creatine (Cr) correlated strongly between LCModel and vendor‐supplied software in the BG. For shorter TEs, the ratios of NAA/Cr and Cho/Cr were more closely related using STEAM at TE = 20 ms in BG and WM, which was significantly better than using PRESS at TE = 30 ms in the BG of HIE infants.

At short TEs, however, it is still unclear which MRS sequence, STEAM or PRESS, is superior and thus more work is required in this regard for translating research‐generated MRS ratios to clinical diagnosis and prognostication, and unlocking the potential of MRS for in vivo metabolomics. MRS at both long and short TEs is desirable for standard metabolites such as NAA, Cho and Cr, along with important lower concentration metabolites such as myo‐inositol and glutathione.

**Abbreviations used**
BG: basal gangli
Cho: total choline
Cr: total creatine
FWHM: full‐width half max
GA: gestational age
GABA: gamma‐Aminobutyric acid
GPC: glycerol‐phosphocholine
GSH: glutathione
HIE: hypoxic ischemic encephalopathy
Lac: lactate
mIns: myo‐inositol
MRS: magnetic resonance spectroscopy
NAA: N‐acetylaspartate
NVD: N‐acetylcysteine and vitamin D
PCh: phosphocholine
PRESS: point‐resolved spectroscopy
PT: preterm
SNR: signal‐to‐noise ratio
STEAM: stimulated acquisition mode
TE: echo time
TR: repetition time
WM: white matter

## 1 INTRODUCTION

In research studies, magnetic resonance spectroscopy (MRS) metabolite ratios are reliable early predictors of developmental outcome following moderate to severe neonatal hypoxic–ischemic injury at term gestational age (GA).^1^, ^2^, ^3^ Biomarkers such as N‐acetylaspartate (NAA), total creatine (Cr), total choline (Cho) and myo‐inositol (mIns) can signify normal or abnormal development, while others such as lactate (Lac) can help assess the degree of acute injury.^4^ In neonatal hypoxic ischemic encephalopathy (HIE), a meta‐analysis has shown that MRS ratios of NAA and Lac in the subacute period after injury provide the best prognostication for long‐term outcome.^1^ In preterm (PT) infants, NAA/Cho and Cho/Cr ratios in the periventricular white matter (WM) at term GA are better predictors of motor outcomes than conventional MRI at one‐year follow‐up.^5^ In the clinical setting, neuroradiologists use the gross relationship of metabolite peak areas quantified using the vendor‐supplied MRS processing software at the scanner console. The ratios of peak metabolite areas derived from vender‐supplied software have neither been validated in neonates nor have they been related to values obtained by more advanced spectral fitting methods in research trials.

MRS is only useful as a noninvasive biomedical imaging tool if results are reliable and reproducible in a clinical setting over the range of values that reflect subject variability.^6^ The burden falls on the community of MRS researchers and clinicians to address the issue of reproducibility of metabolite ratios between research and clinical MRS scans in order to move the field forward.^7^ Short TE spectral fits suffer from multiple overlapping peak shapes that may act as confounds in the final summation of the individual line shapes. Although quite noisy, at long TEs, the fits are more robust to the higher concentration metabolites such as NAA (2.1 ppm), Cho (3.2 ppm) and Cr (3.0 ppm), known for their neuronal, membrane and metabolic indications, respectively, as well as the complete absence of the macromolecule‐lipid signal contribution below 1.3 ppm. Furthermore, barring the SNR differential, the main trade‐off between short and long TEs is the total number of metabolites that can be reliably quantified. Many of the smaller concentration metabolites such as glutathione (GSH, 2.95 ppm) and gamma‐Aminobutyric acid (GABA) (1.9–3 ppm) are dephased at long TEs. Furthermore, these low concentration metabolites are of great interest but have multiple small peak resonances that are usually hidden at short TEs due to the different hydrogen resonances present within the molecules that are swamped by other major metabolites (eg NAA), making them difficult to parse out with any confidence.

One of the most widely used postprocessing programs for quantifying MRS data in research settings is LCModel.^8^, ^9^ LCModel is a proprietary offline, research‐based, spectral analysis program that estimates metabolite peak shapes while minimizing data residuals by using a priori metabolite basis spectra. Reliability of voxel placement, FWHM, SNR, as well as between‐subject and between‐session reproducibility using LCModel, have been addressed in other studies.^10^ LCModel, however, is not currently available for turn‐key spectral quantification online at the clinical MRI scanner console.

Vendor‐supplied software quantifies fewer and less complex spectral peaks than research‐based programs like LCModel. The standard metabolites used to fit proton spectra with the vendor‐supplied software consist of NAA, Cr, Cho, Lac, and mIns. Customized metabolite shapes can be built; however, this is not a practical approach in clinical practice and creates a new problem in the subjectivity of parameter selections. LCModel uses a more complex basis set that includes a significantly larger number of metabolites; most are lower concentration moieties (eg glutathione, γ‐aminobutyric acid, taurine, aspartate, mIns, scyllo‐inositol, glutamine) that resonate near the bases of large concentration peaks or have multiplet peak shapes spread across the frequency spectrum. In addition, LCModel simulates the broad macromolecule and lipid resonances that are situated near the peak resonances of Lac near 1.3 ppm. It is well documented that many less complex fitting models cannot properly handle the numerous in vivo confounds from moieties not accounted for in spectral processing without using the prior knowledge of these moieties as constraints to the fitting algorithm.^11^ These differences in fitting algorithms can lead to significant variability in metabolite ratios, yet few reports systematically compare metabolite ratios from vendor‐supplied processing software and LCModel. One abstract has compared Phillips 1.5 T MRS scanner software metabolite ratios with LCModel outputs at long TEs.^12^ Similar data do not exist for Siemens 3 T MR systems, and to our knowledge no comparisons have been published on neonates.

This comparison is critical to the advancement of MRS as a clinical prognostic tool, not only for disease, injury, recovery and outcome, but also in unlocking the potential of MRS for in vivo metabolomics. Therefore, we present here an investigation into the consistency and reliability of metabolic ratios quantified using vendor‐supplied console‐based MRS fitting software in comparison with the research tool, LCModel, using MRS data obtained on Siemens 3 T TIM Trio and 3 T Skyra MRI scanners (Siemens Healthineers, Erlangen, Germany) in PT infants as well as near‐term and term HIE neonates.

## 2 METHODS

### 2.1 Participants

Neonates and infants were enrolled in three prospective studies with neuroimaging components between August 2013–April 2017 (Table 1). Consent was obtained, and all studies were approved by the Institutional Review Board at the Medical University of South Carolina. A total of 55 infants were scanned on two different 3 T Siemens systems: 15 neonates with HIE (GA = 34–40 weeks) received 72 hours of therapeutic hypothermia (33°C) for moderate to severe HIE and were scanned at 4–8 days on the Siemens 3 T Skyra; 27 HIE neonates (GA > 35 weeks), enrolled in a study of N‐acetylcysteine and vitamin D (NVD) in addition to hypothermia, were scanned at 5–6 days on the clinical 3 T Skyra, 19 of whom returned for an unsedated scan at 2–5 weeks after birth on a research Siemens 3 T TIM Trio; 13 PT infants, who were 27–32 weeks GA at birth and enrolled in a nonintervention trial, were scanned unsedated at term age equivalent (GA = 36–44 weeks) on the TIM Trio. A total of 42 complete datasets were obtained on the Siemens 3 T Skyra, and 32 scans on the Siemens 3 T TIM Trio.

**Table 1:** Patient demographics

Study	No. of patients	No. of scans	GA at birth (wk)	GA at scan (wk)
HIE	15	15	34–40	34–41
ΗΙΕ + ΝVD	27	46	>35	27 at 35–36 19 repeats at 37–41
Noninterventional trial	13	13	27–32	40–44

### 2.2 MRS protocols

Single‐voxel point‐resolved spectroscopy (PRESS) MRS was performed in the basal ganglia (BG) and frontal WM [TE = 30 ms and 270 ms, TR = 2000 ms, voxel size = (3.375–8 mm)^3^ (median = {3.375 mm}^3^) isotropic, number of averages = 100–192 (median_30TE_ = 128; median_270TE_ = 192), BW = 1200 Hz, flip angle = 90 degrees, nominal refocusing flip angle = 180 degrees]. For 27 HIE neonates, single‐voxel stimulated acquisition mode (STEAM) MRS [TE = 20 ms, TR = 1500 or 2000 ms, voxel size = (3.375–8 mm)^3^ (median = {8 mm}^3^), number of averages = 64–256 (media*n* = 176), BW = 1200 Hz, flip angle = 90 degrees, nominal refocusing flip angle = 180 degrees] was also performed in the left BG and frontal WM area at multiple time points around the NVD infusion on day of life 5–6 and at the follow‐up scan (2 weeks to 2 months).

### 2.3 Spectral processing

Versatile Simulation, Pulses, and Analysis (VeSPA)^13^ was used to generate customized basis sets, as previously described,^14^ at all TEs while adhering to standard LCModel experimental basis set parameters. Calibration of N‐Acetylaspartylglutamic acid (NAAG) to NAA and GPC to PCh was then performed once the simulations were finished and the basis sets finalized (as outlined in detail in the LCModel manual). Next, all subjects' spectra were processed using these simulated basis sets with LCModel.

**Figure 1:**
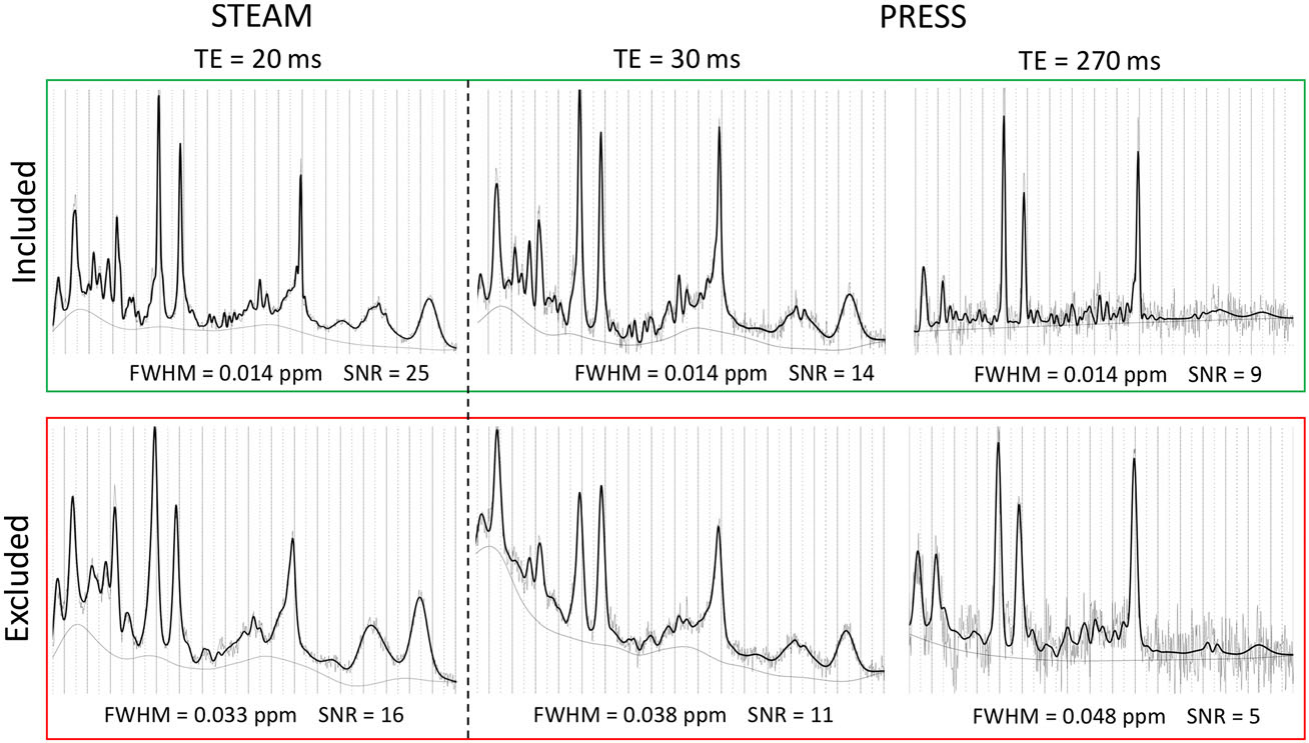
Example MRS spectra from the three different echo times (TE) used in this study. The spectra framed by the green box are those which met both inclusion criteria of a full‐width‐half‐max (FWHM) < = 0.03 ppm and signal‐to‐noise‐ratio (SNR) > 5; those outlined by the red box show representative samples of excluded spectra. The thin gray line is the raw data and the solid black line is the LCModel fit; the smooth thin gray line below the raw data is the baseline generated automatically by LCModel prior to quantifying each metabolite peak

Anonymized Siemens MRS RDA files were processed using LCModel. Eddy‐correction was performed for all MRS scans using the built‐in functionality of LCModel. The processed spectra were then viewed by a physicist (Dr. Brown) for quality assurance, and the FWHM and SNR were evaluated for meaningfulness of metabolite ratios. All those which were of sufficient quality were included in the database. To obtain only the highest quality scans, inclusion criteria for processed MRS were based on spectral quality as reported by LCModel (FWHM ≤0.03 and SNR > 5), as well as for obvious artifacts due to gross motion and poor water suppression. Representative spectra can be seen in Figure 1.

### 2.4 Data analysis

Because absolute quantification is not currently available with the vendor‐supplied software, and since it is standard practice for most clinicians to report their findings relative to the Cr peak, LCModel metabolite ratios relative to Cr were used for our analysis. The manufacturer indicated that the same processing software was used in each of the TIM Trio and Skyra platforms (personal communication), and our comparison between vendor‐supplied program outputs verified similar ratios; therefore, we grouped the data from the two Siemens MR systems for the comparison with LCModel.

The LCModel processing algorithm takes into account the difference in the number of protons between Cr (3) and Cho (9) during its calculations, whereas the vendor‐supplied software does not. Thus, we divided the calculated Cho/Cr ratio from the Siemens software by 3 to normalize the analysis for comparisons with LCModel.

Metabolite ratios were excluded from analysis if the standard deviations were > 20% for NAA/Cr, Cho/Cr or mIns/Cr, as Cramer‐Rao lower bounds reported by LCModel. The outputs were correlated with Spearman or Pearson correlation coefficients as appropriate, depending on the distribution of the data within each cohort (HIE or PT) and brain region (BG or WM).

Finally, when metabolite ratios from LCModel and vendor‐supplied fitting software were significantly correlated in both STEAM and PRESS sequences (in the BG of HIE infants), we performed a Fisher's r‐to‐z transformation to determine whether the differences in strength of correlations were significant between the two sequences. For metabolites whose correlations were significant using one but not the other sequence, for instance in the WM spectra, then the transformation was unnecessary as the superior sequence was already known.

## 3 RESULTS

Demographics of our MRS cohorts are detailed in Table 1. All scans were conducted between 36–48 weeks GA, and included neonates and infants with a variety of clinical conditions and stages of injury. Our data reflect this significant intra‐ and inter‐subject heterogeneity, with variations in the metabolite ratios between WM and BG, degree of acute injury in HIE infants, as well as developmental changes in convalescing HIE and PT infants. Our analysis, therefore, provides a broad range of ratios reflecting the spectrum of neonatal disease and several months of early infant development.

**Figure 2:**
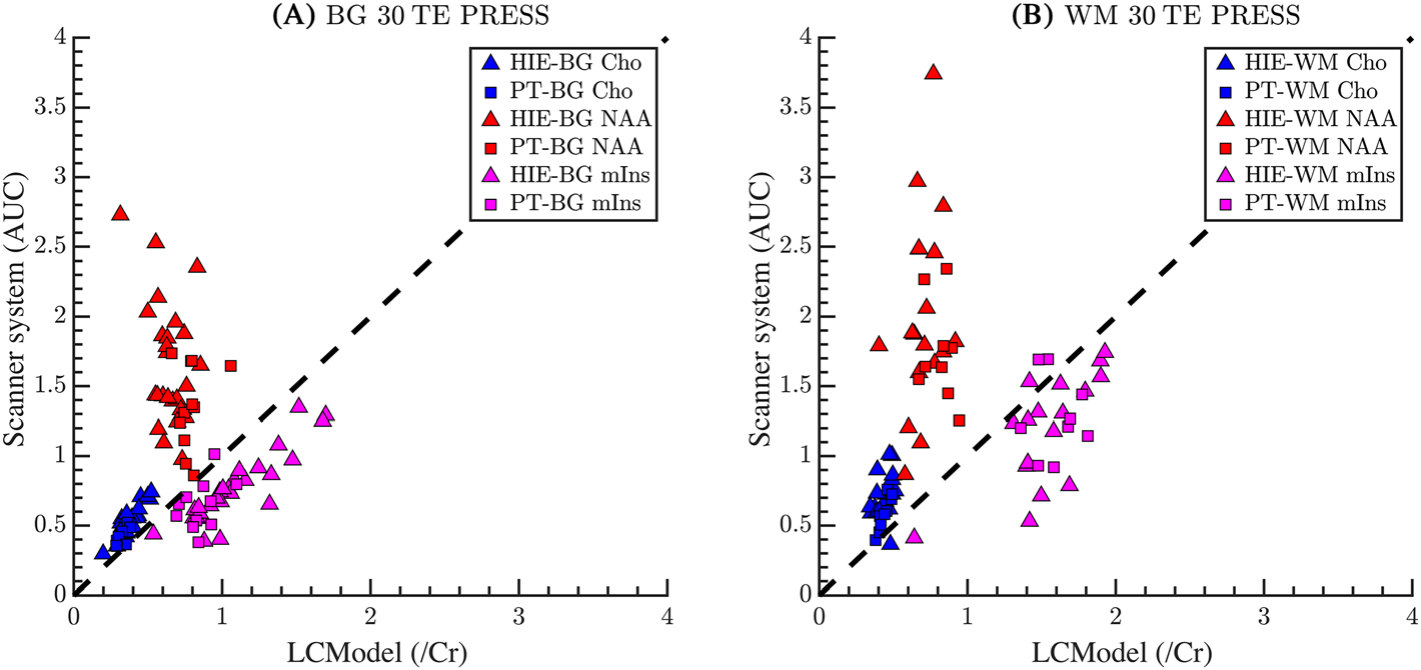
Comparing metabolite ratios between the two methods at TE = 30 ms using a PRESS MRS sequence for (A) BG and (B) WM. The y‐axis shows the vendor‐supplied scanner software output values and the x‐axis shows the values from LCModel. All values reported by the scanner and LCModel are ratios to Cr. The dashed line is the line of unity. Reasonable agreement can be seen for Cho and mIns in both the BG and WM. However, there is a greater range of NAA concentrations from the scanner compared with LCModel for both brain tissue regions. The reason for the large spread in NAA values reported from the console is not clear

At TE = 30 ms using PRESS, n_total_ = 138 spectra due to repeated scans in individual subjects, and of those, 64 spectra met the strict inclusion criteria. The relationship between vendor‐supplied and LCModel outputs varied by metabolites (Figure 2A and B). There is a clear association for Cho/Cr ratios in both cohorts in the BG, but only for the PT cohort in WM; mIns/Cr ratios correlate in both BG and WM in HIE infants, but not in PT infants. Most importantly, the vendor‐supplied software overestimated NAA/Cr with large variability, while the LCModel outputs for NAA/Cr ratios appear to be relatively stable. Correlation coefficients and the corresponding *p*‐values are presented in Table 2.

**Table 2:** TE = 30 ms, PRESS (n = 64)

Region	Basal ganglia (BG)				Frontal water shed (WM)			
Condition	HIE (n = 27)		PT (n = 11)		HIE (n = 17)		PT (n = 9)	
Metabolite	r	p	r	p	r	p	r	p
Cho	0.90	<0.01*	0.71	0.01*	0.34	0.18	0.91	<0.01*
NAA	−0.40	0.04*	0.18	0.59	0.32	0.21	−0.25	0.51
mIns	0.89	<0.01*	0.41	0.21	0.71	<0.01*	−0.04	0.93

* *p* < 0.05.

**Figure 3:**
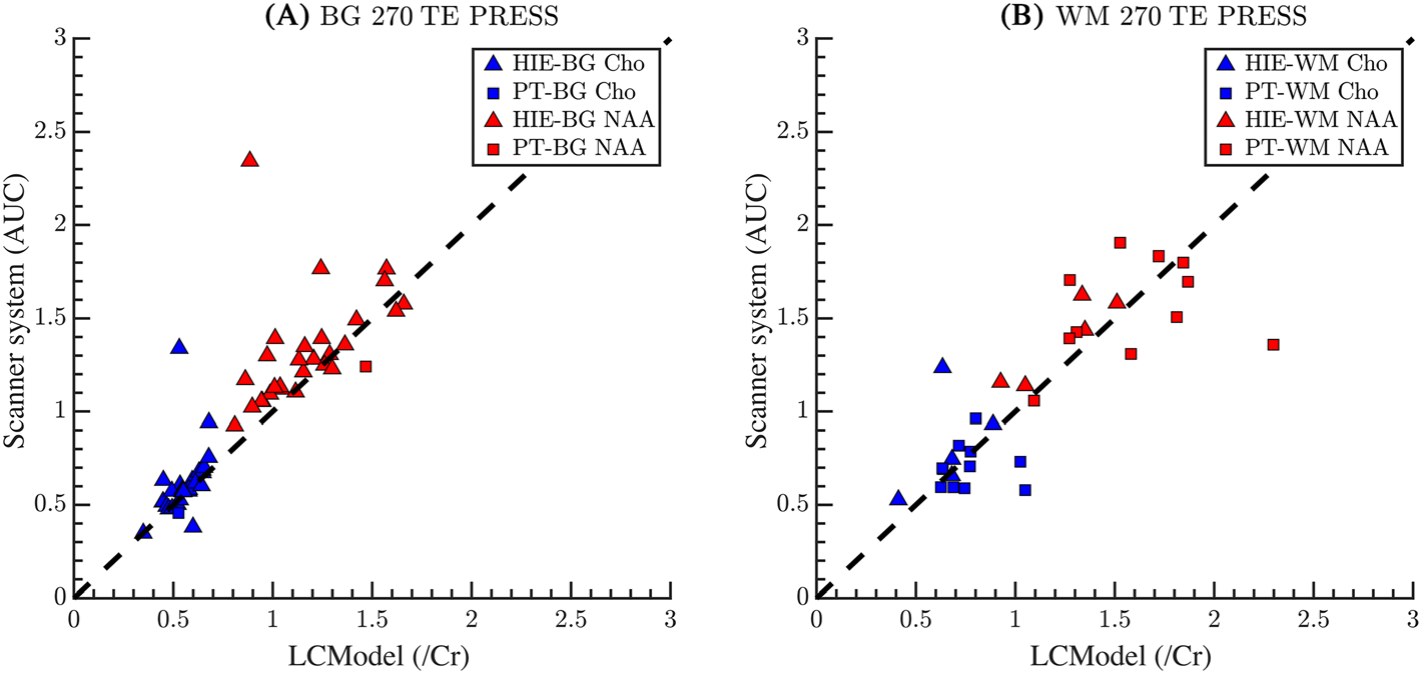
Comparison of metabolite ratios between the vendor‐supplied spectral fitting software and LCModel at TE = 270 ms using a PRESS MRS sequence for (A) BG and (B) WM. The y‐axis shows the console outputs while the x‐axis shows the reported values from the LCModel. All values plotted are reported as relative to Cr. There exists a strong agreement between the methods for both Cho and NAA in the BG and WM. Note that at this longer TE, mIns is no longer quantifiable. The dashed line represents the line of unity. The tighter agreement of NAA seen here compared with shorter TE may be the result of minimal peak shape overlap that improves initial fitting conditions

At the longer TE of 270 ms, n_total_ = 131 scans due to repeated scans, with 45 useable spectra that met the inclusion criteria. We found that the calculated ratios of NAA/Cr and Cho/Cr correlated strongly between LCModel and vendor‐supplied software (Figure 3A and B). Cho/Cr is reliably correlated between software outputs in the BG in HIE infants, but not in WM for either cohort; mIns was not compared, as it is not reliably measured at TE = 270 ms. Correlation coefficients and *p*‐values are presented in Table 3.

**Table 3:** TE = 270 ms, PRESS (n = 45)

Region	Basal ganglia (BG)				Frontal water shed (WM)			
Condition	HIE (n = 28)		PT (n = 1)		HIE (n = 5)		PT (n = 11)	
Metabolite	r	p	r	p	r	p	r	p
Cho	0.67^#^	<0.01*	N/A	N/A	0.40^#^	0.52	0.18^#^	0.60
NAA	0.45	0.02*	N/A	N/A	0.60^#^	0.35	0.24^#^	0.49

* *p* < 0.05, ^#^Spearman's *r*.

**Figure 4:**
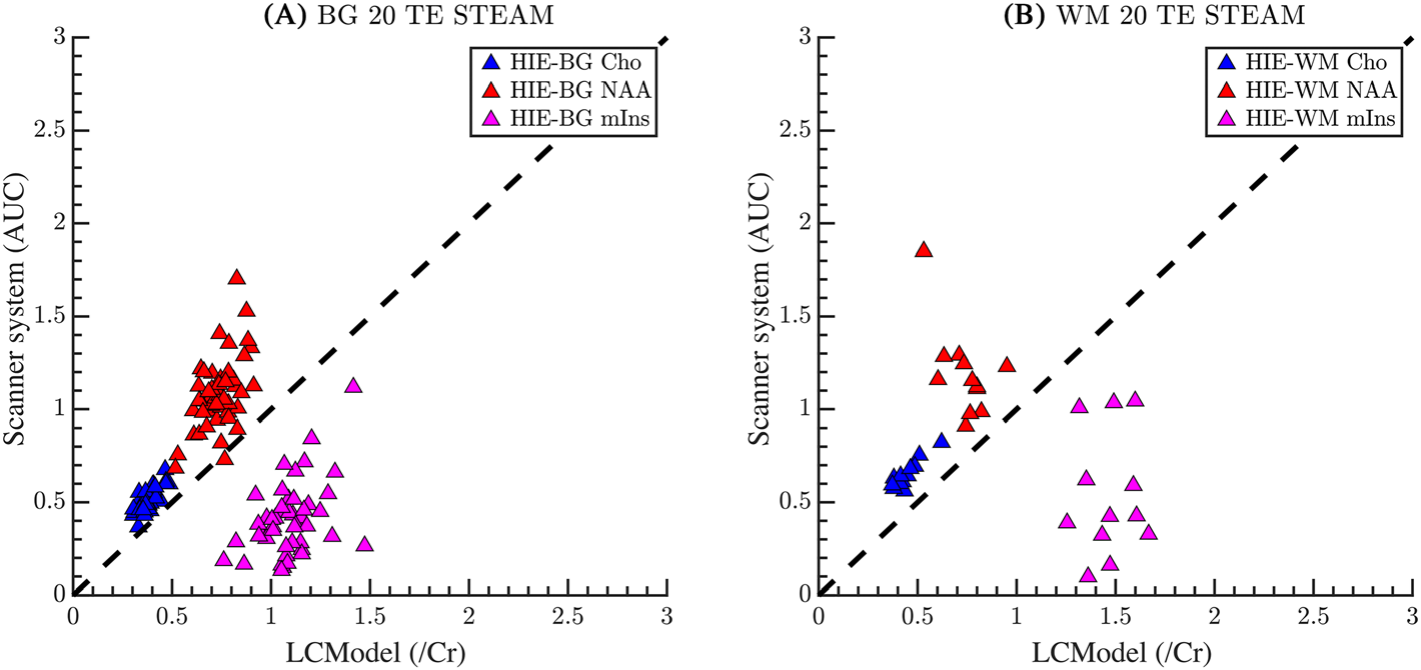
Comparison of metabolite ratios at TE = 20 ms using a STEAM MRS sequence for (A) BG and (B) WM. The y‐axis shows the vendor‐supplied spectral fitting output and the x‐axis shows the output values from LCModel. The dashed line represents the line of unity. All values are ratios relative to Cr. Both BG and WM regions have relative agreement between the console fitting and the LCModel for Cho and NAA. For mIns, the scanner console underestimates the concentrations compared with the LCModel although the estimates are grouped rather coherently. This suggests that a simple scaling factor may be applied to mIns to improve the agreement

Using STEAM at TE = 20 ms, n_total_ = 128 scans with repeated scans, primarily on HIE infants of GA > 35 weeks. Of these, 59 spectra met the inclusion criteria with only one from the PT group located in the BG region. Therefore, we included this PT scan in the HIE category for correlation within either region, BG or WM. The ratios of NAA/Cr are closely related between software analyses in BG and WM (Figure 4A and B), and show a much less severe systematic mismatch between software outputs than with the PRESS at TE = 30 ms sequence. Cho/Cr ratios are also significantly correlated in both BG and WM; mIns/Cr shows a weak relationship between the two outputs with the ratio being underestimated by the vendor‐supplied software in relation to LCModel estimations. Correlation coefficients and *p*‐values for TE = 20 ms STEAM are presented in Table 4.

**Table 4:** TE = 20 ms, STEAM (n = 59)

Region	Basal ganglia (BG)		Frontal water shed (WM)	
Condition	HIE (n = 27)	PT (n = 11)	HIE (n = 17)	PT (n = 9)
Metabolite	r	p	r	p
Cho	0.77	<0.01*	0.91	<0.01*
NAA	0.54	<0.01*	−0.59	0.05*
mIns	0.42	0.01*	0.07	0.84

* *p* < 0.05.

Comparing ratios between PRESS TE = 30 ms (Figure 2) and STEAM TE = 20 ms (Figure 4) for the HIE cohort, correlation of software outputs for NAA/Cr ratios are significantly stronger using short echo STEAM in both BG and WM, and for Cho/Cr ratios in the WM rather than PRESS; mIns/Cr agreement is underestimated by both sequences in the BG, but is better with PRESS TE = 30 ms in the WM.

The calculated correlation coefficients of software outputs in the BG using STEAM at TE = 20 ms were compared with PRESS at TE = 30 ms or PRESS at TE = 270 ms in HIE patients who had all three sequences performed (Table 5). For NAA/Cr, the correlation is significantly higher for TE = 20 ms STEAM than for TE = 30 ms PRESS, and NAA/Cr correlations between software fitting of TE = 20 ms STEAM were not significantly different to those using TE = 270 ms PRESS. For Cho/Cr, correlations using STEAM and PRESS sequences are not significantly different. For mIns/Cr, the correlation between software metabolite ratios is better for PRESS at TE = 30 ms than for STEAM at TE = 20 ms.

**Table 5:** Fischer's r‐to‐z transformation in BG HIE subjects

Metabolite (/Cr)	STEAM 20 ms	PRESS 30 ms	PRESS 270 ms	20 vs. 30		20 vs. 270	
	r	r	r	z	p	z	p
Cho	0.77	0.90	0.67	−1.78	0.08	0.84	0.40
NAA	0.54	−0.40	0.45	4.05	<0.01*	0.48	0.63
mIns	0.42	0.89	N/A	−3.84	<0.01*	N/A	N/A

* *p* < 0.05.

## 4 DISCUSSION

With MRS becoming more widely used as a measure of acute brain injury as well as a prognostication tool, clinical neuroradiologists need reliable metabolite peak area ratios which do not require special postprocessing software. Therefore, we tested if the standard Siemens 3 T MRS fitting software quantifies individualized patient ratios comparable with those obtained by analysis with LCModel, which is one of the most widely used spectral fitting software programs used in research studies. Our results indicate that at a longer TE (ie 270 ms) in a diverse group of neonates, NAA/Cr and Cho/Cr ratios are in excellent agreement between embedded vendor‐supplied and LCModel software fitting results. We investigated two standard clinically available short TE sequences routinely used to quantify metabolites having more complex spectra or that are present in lower concentrations. The STEAM sequence at TE = 20 ms produced NAA/Cr and Cho/Cr ratios that were strongly correlated between the two software programs. However, with PRESS at TE = 30 ms there was poor agreement between the NAA/Cr ratios obtained with the vendor‐supplied and LCModel spectral processing. With both short TE sequences, scanner software appears to underestimate the mIns/Cr ratio, which could roughly be alleviated with a simple intercept shift. This discrepancy between the fitting methods most likely stems from the fact that LCModel accounts for the dephasing of the coupled peaks of mIns in the basis set, whereas the scanner software does not. The simple peak area ratios of the scanner are not robust enough to capture this dephasing and therefore underestimate the true values.

At short TEs, overlapping metabolite peaks create spectral complexity in vivo and may contribute to the differences in metabolite ratios that we observed, particularly for NAA/Cr. Peak structure around NAA is highly complex at short TEs, leading to results which are not only sensitive to the spectral fitting, but also to background subtraction and the fit of potential confounding molecules. NAA is a valuable reference metabolite, as it correlates well with healthy neurons and developmental outcomes, and Cr measures cellular energetics.^2^, ^15^ As investigators commonly use ratios which have NAA or Cr as the denominator, we also investigated the relationship of NAA/Cr between software fitting programs using the STEAM and PRESS sequences. NAA/Cr ratios are considerably better correlated in the STEAM rather than PRESS sequences at short TEs, although it is still unclear as to why this is the case. In addition, PRESS Cho/Cr ratios at the long TE in neonatal WM were not correlated between the two analysis methods. Futhermore, PRESS at short TEs showed poor agreement between the fitting methods for Cho/Cr in WM. Therefore, caution should be taken when using these short TE MRS sequences without other complementary means of validation for diagnostic purposes.

Both Cr and NAA have been shown to be in flux after stroke,^2^, ^16^ and quantifying both as ratios would be valuable in HIE infants, as they measure different metabolic pathways. Referencing metabolite concentrations of NAA and Cr to Cho (ie NAA/Cho or Cr/Cho) may be an alternative at short TEs, for the purpose of comparison with research data,^5^, ^17^ as Cho appears to be less confounded, perhaps due to relatively sparse contributions to the peak shape by any other resonances within its vicinity. In addition, it would be desirable to relate one changing metabolite to a more stable one, such as Cho, after acute HIE injury.^2^

Cho and Cr have large amplitudes and consistent peak shapes, and the Cho/Cr ratio correlated well in PT and term groups using both short TE sequences, with the exception of PRESS outputs from the frontal WM in HIE infants, which did not correlate between LCModel and vendor‐supplied software; the mIns/Cr ratio was also underestimated by the vendor‐supplied software. Therefore, our data indicates that for those metabolites best imaged at shorter TEs due to better SNR, the increased complexity of spectra requires care in sequence selection as it is still unclear which short TE sequence is superior and implies that further studies are needed. We speculate that if the vendor‐supplied fitting algorithm included peak shapes from all the available metabolites utilized by LCModel fitting then the vendor‐supplied and LCModel fits would be more comparable. An increased number of scanner peak resonances would potentially act to explain more variance and decrease error in the fit, thereby generating more reliable metabolite estimations. One thing to note is that the frontal watershed WM region of neonates is difficult to shim correctly as it is highly susceptible to nonstationary B‐field fluctuations due to the bone to tissue interface as well as incomplete fat suppression (with PRESS). Shimming may have had an affect on the FWHM and the SNR, and increased the variability of outputs.

At 270 ms TE, most extraneous metabolites (such as mIns, glutathione, etc.) have dephased and the main contributors to the resulting signal are NAA, Cho and Cr, leading to high correlation between the two software outputs. Lac/Cr, though touted as an important biomarker, was not able to be reliably quantified, even using LCModel in most patients. Possible explanations include the transient nature of Lac expression after HIE, and low concentrations otherwise, and overlap with macromolecular peaks making Lac fitting difficult with high standard deviations. Almost every Lac output from LCModel was unuseable, with very few exceptions, so was not included in our analysis due to excessively large Cramer‐Rao bounds, indicating an unsatisfactory fit. One thought was to remove the simulated macromolecule basis set from LCModel, but Dr. Provencher, the creator of LCModel, advised us against this approach (personal communication). Another suggestion is that the line shape used to fit the raw signal may be a significant factor, even if the peak is fit correctly (eg double‐peak, Lorentzian or Gaussian shapes). This factor seems more likely at short TEs rather than long TE spectra, in which nearly all of the macromolecules have accumulated phase and do not contribute to the measured echo signal. Alternatively, the simulated and experimental basis sets may not agree for Lac/Cr. A more recent and promising advancement using diffusion‐weighted MRS was able to reliably separate the overlapping Lac and lipid signals at 1.3 ppm in rat brain tumors.^18^ This technique is not only technically challenging but is still in the early stages of development and is far removed from clinical application. Moreover, utilizing a TE = 144 ms would also mitigate this issue, as is well documented, since the doublet of Lac will invert relative to the other peaks, making for better identification. It is quite possible that a chemical shift displacement with the lipid signals around 1.3 ppm overlapping with the Lac signal is the main culprit for unreliable quantification by either fitting algorithm.^19^ Thus, a possible solution would be to optimize or increase the bandwidth used in future clinical scans. Further investigation into the discrepancy between the simulated and experimental basis sets needs to be undertaken so that in the future Lac/Cr may be compared between both fitting algorithms. More sensitive and accurate quantification of Lac/Cr would be useful clinically in HIE and other metabolic and tumor pathologies.

A strength of our study is the heterogeneity of patients and gestational ages scanned. As biomarkers, the metabolite ratios should differ among individual patients, as NAA/Cr ratios have been shown by many investigators to correlate with outcome after neonatal HIE and PT birth.^1^, ^20^, ^21^, ^22^ Figures 2, 3, 4 illustrate the expected heterogeneity of metabolite ratios in our two cohorts in the BG and WM by disease type. We included cohorts of term infants with brain injury and infants born prematurely, as these groups are expected to undergo MR imaging and may benefit from MRS as part of clinical prognostication for outcome. As expected, term infants scanned within a week of acute, hypoxic ischemic brain injury have dramatically different ratios than when scanned 2–8 weeks later, as do PT infants scanned 8–12 weeks after birth, who may have experienced more chronic diffuse cerebral insults. In addition, ratios differed in WM and BG in both groups, perhaps reflecting different susceptibilities to injury, different rates of maturation with development, as well as overall differences in metabolism. As development proceeds, the metabolite variability also reflects different GA at scan as well as different disease processes.^5^, ^22^, ^23^, ^24^, ^25^, ^26^ We believe the heterogeneity of measured ratios adds to, rather than detracts from, the generalizability of our comparison data.

Our study's limitations include using only a single vendor‐supplied software program for comparison with LCModel's outputs. At our institution we exclusively have Siemens scanners, but other studies are underway utilizing other manufacturers' 3 T systems, which will add to our data on reliability and harmonization across different platforms (MARBLE, NCT01309711). It is our hope that these studies will ultimately facilitate the translation of MRS to clinical practice as a prognostic modality in neonates, as well as using MRS as intermediate outcomes for clinical trials.^27^, ^28^ Further experimentation may validate our initial findings with more rigorous experimental constraints. We hope to work closely with the MR vendors to improve the quantitation of their supplied fitting software for more reliable metabolite ratios. Furthermore, the ability to quantify absolute molar concentrations of these intra‐cellular metabolites at the scanner console would be a great step towards better understanding of in vivo metabolomics in different disease states and clinical outcomes.

Neonates represent a complex and challenging population as prognostication techniques must address deviations from normal development, changes due to injury, and the interaction of age of development with injury. Nevertheless, this population has a great need for noninvasive neuroimaging assessments, as diverse therapies may be instituted based on neuroimaging results before developmental delays manifest and become fixed developmental impairments. Therefore, we are of the opinion that the use of MRS and other imaging modalities may improve outcomes by identifying high‐risk infants, tracking progress with therapies, and influencing neuroplasticity during an important time in development, all of which can impact upon clinical management. As MRS is a useful prognostic tool in research studies,^29^ clinical correlation studies such as ours must proceed in parallel with support from MR vendors, to be able to translate the research advances in MR neuroimaging towards better clinical utility and acceptance.

## 5 CONCLUSION

MRS metabolite results for NAA/Cr and Cho/Cr ratios using PRESS at TE = 270 ms appear robust and comparable by different analytical methods currently in use in neonates undergoing research and clinical neuroimaging. As metabolites that contribute to the signal at longer TEs are sparse, neuroradiologists reading vendor‐supplied spectral scans can be confident in their diagnostics involving Cr, NAA and Cho ratios in the BG at TE = 270 ms. Short TE spectral methods, with more metabolites to quantify, show promise but need more work to better understand the underlying processes that are in disagreement between the vendor‐supplied software and the LCModel fitting algorithms. STEAM at TE = 20 ms offers better agreement in NAA/Cr and Cho/Cr ratios than short echo PRESS at TE = 30 ms, while mIns/Cr appears to be related between LCModel and the vendor‐supplied software, but may require a possible correction factor. To show MRS is useful clinically as a biomarker for neonatal brain injury and abnormal development, further multi‐institutional trials are needed to expand and enhance online scanner analysis compared with offline software analysis, as we have presented for Siemens systems.

## FUNDING INFORMATION

MUSC Neuroscience Institute grant South Carolina Clinical & Translational Research Institute UL1 TR000062. National Institute for Health (NIH) Research grant (to J. Dubno), Grant/Award Number: T32DC0014435; National Institute for Health (NIH) Research grant (to N. Demore), Grant/Award Number: T32GM008716; South Carolina Clinical & Translational Research Institute.

## References

[1] Thayyil S , Chandrasekaran M , Taylor A , et al. Cerebral magnetic resonance biomarkers in neonatal encephalopathy: a meta‐analysis. Pediatrics. 2010;125(2):e382‐e395. 20083516 10.1542/peds.2009-1046

[2] Cheong JL , Cady EB , Penrice J , Wyatt JS , Cox IJ , Robertson NJ . Proton MR spectroscopy in neonates with perinatal cerebral hypoxic–ischemic injury: metabolite peak‐area ratios, relaxation times, and absolute concentrations. AJNR Am J Neuroradiol. 2006;27(7):1546‐1554. 16908578 PMC7977542

[3] Alderliesten T , de Vries LS , Benders MJ , Koopman C , Groenendaal F . MR imaging and outcome of term neonates with perinatal asphyxia: value of diffusion‐weighted MR imaging and 1 H MR spectroscopy. Radiology. 2011;261(1):235‐242. 21828190 10.1148/radiol.11110213

[4] Xu D , Vigneron D . Magnetic resonance spectroscopy imaging of the newborn brain–a technical review. Semin Perinatol. 2010;34(1):20‐27. 20109969 10.1053/j.semperi.2009.10.003 PMC2842012

[5] Kendall GS , Melbourne A , Johnson S , et al. White matter NAA/Cho and Cho/Cr ratios at MR spectroscopy are predictive of motor outcome in preterm infants. Radiology. 2014;271(1):230‐238. 24475798 10.1148/radiol.13122679

[6] Kreis R . Quantitative localized 1 H MR spectroscopy for clinical use. Prog Nucl Mag Res Sp. 1997;31(2):155‐195.

[7] Schirmer T , Auer DP . On the reliability of quantitative clinical magnetic resonance spectroscopy of the human brain. NMR Biomed. 2000;13(1):28‐36. 10668051 10.1002/(sici)1099-1492(200002)13:1<28::aid-nbm606>3.0.co;2-l

[8] Provencher SW . Estimation of metabolite concentrations from localized in vivo proton NMR spectra. Magn Reson Med. 1993;30(6):672‐679. 8139448 10.1002/mrm.1910300604

[9] Provencher SW . Automatic quantitation of localized in vivo 1H spectra with LCModel. NMR Biomed. 2001;14(4):260‐264. 11410943 10.1002/nbm.698

[10] Fayed N , Modrego PJ , Medrano J . Comparative test–retest reliability of metabolite values assessed with magnetic resonance spectroscopy of the brain. The LCModel versus the manufacturer software. Neurol Res. 2009;31(5):472‐477. 19215666 10.1179/174313209X395481

[11] Mierisova S , Ala‐Korpela M . MR spectroscopy quantitation: a review of frequency domain methods. NMR Biomed. 2001;14(4):247‐259. 11410942 10.1002/nbm.697

[12] Chu Z , Wang ZJ , Chia J , Hunter JV . Comparison of Quantification of Clinical MR Spectra by LCModel and the Scanner System Software. Paper presented at Proceedings of the International Society of Magnetic Resonance in Medicine, 2007, Berlin, Germany.

[13] Soher BJ , Semanchuk P , Todd D , Steinberg J , Young K . Vespa: Integrated applications for RF pulse design, spectral simulation and MRS data analysis. Paper presented at Proceedings of the International Society of Magnetic Resonance in Medicine 2011, Quebec, Canada. 10.1002/mrm.29686 PMC10330446 37183778

[14] Moss HG , Brown TR , Wiest DB , Jenkins DD . N‐Acetylcysteine rapidly replenishes central nervous system glutathione measured via magnetic resonance spectroscopy in human neonates with hypoxic–ischemic encephalopathy. J Cereb Blood Flow Metab. 2018:271678X18765828, 38(6):950‐958. 10.1177/0271678X18765828 PMC5999009 29561203

[15] Rae CD . A guide to the metabolic pathways and function of metabolites observed in human brain 1H magnetic resonance spectra. Neurochem Res. 2014;39(1):1‐36. 24258018 10.1007/s11064-013-1199-5

[16] Gano D , Chau V , Poskitt KJ , et al. Evolution of pattern of injury and quantitative MRI on days 1 and 3 in term newborns with hypoxic–ischemic encephalopathy. Pediatr Res. 2013;74(1):82‐87. 23618911 10.1038/pr.2013.69

[17] Johnson CB , Jenkins DD , Bentzley JP , et al. Proton magnetic resonance spectroscopy and outcome in term neonates with chorioamnionitis. J Perinatol. 2015;35(12):1030‐1036. 26426253 10.1038/jp.2015.121 PMC4660057

[18] Wang AM , Leung GK , Kiang KM , Chan D , Cao P , Wu EX . Separation and quantification of lactate and lipid at 1.3 ppm by diffusion‐weighted magnetic resonance spectroscopy. Magn Reson Med. 2017;77(2):480‐489. 26833380 10.1002/mrm.26144

[19] Kreis R . Issues of spectral quality in clinical 1H‐magnetic resonance spectroscopy and a gallery of artifacts. NMR Biomed. 2004;17(6):361‐381. 15468083 10.1002/nbm.891

[20] Meyer‐Witte S , Brissaud O , Brun M , Lamireau D , Bordessoules M , Chateil JF . Prognostic value of MR in term neonates with neonatal hypoxic–ischemic encephalopath: MRI score and spectroscopy. About 26 cases. Arch Pediatr. 2008;15(1):9‐23. 18164915 10.1016/j.arcped.2007.08.027

[21] Khong PL , Tse C , Wong IY , et al. Diffusion‐weighted imaging and proton magnetic resonance spectroscopy in perinatal hypoxic–ischemic encephalopathy: association with neuromotor outcome at 18 months of age. J Child Neurol. 2004;19(11):872‐881. 15658792 10.1177/08830738040190110501

[22] Chau V , Brant R , Poskitt KJ , Tam EW , Synnes A , Miller SP . Postnatal infection is associated with widespread abnormalities of brain development in premature newborns. Pediatr Res. 2012;71(3):274‐279. 22278180 10.1038/pr.2011.40 PMC3940469

[23] Akasaka M , Kamei A , Araya N , et al. Assessing Temporal Brain Metabolite Changes in Preterm Infants Using Multivoxel Magnetic Resonance Spectroscopy. Magn Reson Med Sci. 2016;15(2):187‐192. 26567757 10.2463/mrms.mp.2015-0041 PMC5600055

[24] Bluml S , Wisnowski JL , Nelson MD Jr , Paquette L , Panigrahy A . Metabolic maturation of white matter is altered in preterm infants. PLoS One. 2014;9(1):e85829. 24465731 10.1371/journal.pone.0085829 PMC3899075

[25] Tanifuji S , Akasaka M , Kamei A , et al. Temporal brain metabolite changes in preterm infants with normal development. Brain Dev. 2017;39(3):196‐202. 27838187 10.1016/j.braindev.2016.10.006

[26] Kreis R , Ernst T , Ross BD . Development of the human brain: in vivo quantification of metabolite and water content with proton magnetic resonance spectroscopy. Magn Reson Med. 1993;30(4):424‐437. 8255190 10.1002/mrm.1910300405

[27] Lally PJ , Pauliah S , Montaldo P , et al. Magnetic Resonance Biomarkers in Neonatal Encephalopathy (MARBLE): a prospective multicountry study. BMJ Open. 2015;5(9):e008912. 10.1136/bmjopen-2015-008912 PMC4593140 26423856

[28] Azzopardi D , Robertson NJ , Bainbridge A , et al. Moderate hypothermia within 6 h of birth plus inhaled xenon versus moderate hypothermia alone after birth asphyxia (TOBY‐Xe): a proof‐of‐concept, open‐label, randomised controlled trial. Lancet Neurol. 2016;15(2):145‐153. 26708675 10.1016/S1474-4422(15)00347-6 PMC4710577

[29] Spittle AJ , Thompson DK , Brown NC , et al. Neurobehaviour between birth and 40 weeks' gestation in infants born <30 weeks' gestation and parental psychological wellbeing: predictors of brain development and child outcomes. BMC Pediatr. 2014;14(1):111. 24758605 10.1186/1471-2431-14-111 PMC4016657

